# Profiling of the germline mutation *BRCA1*: p.Ile1845fs in a large cohort of Han Chinese breast cancer

**DOI:** 10.1186/s41065-019-0115-7

**Published:** 2019-12-31

**Authors:** Yu Wu, Huanhuan Zhang, Xiaoling Weng, Honglian Wang, Qinghua Zhou, Ying Wu, Yi Shen, Zhen Hu

**Affiliations:** 1 State Key Laboratory of Genetic Engineering and Collaborative Innovation Center for Genetics and Department, School of Life Science, Fudan University, Shanghai, 200436 People’s Republic of China; 2 Department of Biostatistics and Computational Biology, School of Life Sciences, Fudan University, Shanghai, 200436 People’s Republic of China; 3 AITA medical research institute, Shanghai, 200000 People’s Republic of China; 4 Department of Breast Surgery, Fudan University Shanghai Cancer Center, 270 Dongan Road, Xuhui, Shanghai, 200032 China; 5 Department of surgery, Luwan Branch of Ruijin Hospital, Shanghai Jiao Tong University School of Medicine, Shanghai, China

**Keywords:** Breast cancer, *BRCA1*, P.Ile1845fs, Clinicopathological

## Abstract

**Background:**

Breast cancer is a one of the malignant carcinomas partially caused by genetic risk factors. Germline *BRCA1* gene mutations are reportedly associated with breast cancers. Identification of *BRCA1* mutations greatly improves the preventive strategies and management of breast cancer. The aim of our study was to investigate the frequency of the deleterious *BRCA1*: p.Ile1845fs variant in breast carcinomas, as well as the correlation between p.Ile1845fs variant with clinicopathological parameters and clinical outcomes.

**Results:**

A total of 23,481 clinically high-risk patients with breast cancer and 6489 healthy controls were recruited for p.Ile1845fs variant sequencing (either sanger or next generation sequencing). We identified 94 breast cancer patients (0.40%, 94/23481) as well as 11 healthy controls (0.17%, 11/6489) carried p.Ile1845fs variant. *BRCA1*: p.Ile1845fs variant showed a higher frequency in patients with TNBC molecular typing (20.21%, 19/94) and family history (37.23%, 35/94) compared with non-carriers (*P* = 3.62E-6 and 0.034, respectively). According to our data, we advanced the frequency of p.Ile1845fs variant and we confirmed that *BRCA1*: p.Ile1845fs variant was associated with increased risk of breast cancer (OR = 2.36, 95%CI = 1.26–4.89, *P* = 0.004).

**Conclusions:**

*BRCA1*: p.Ile1845fs variant was a frequently pathogenic mutation in breast cancer in Han Chinese women and our data may be helpful for diagnosis and therapy of breast cancer.

## Background

Breast cancer is a leading health concern among women worldwide, with approximately 252,710 women newly diagnosed cases occurring every year in the world [1, 2]. In the past few years, the rates of mortality have decreased as a result of recent advancements in the understanding of breast cancer biology. However, breast cancer still keeps up with the leading cause of death in women and metastases at distant sites are still responsible for majority of the cancer death [3]. The genetic architecture of breast cancer involves germline pathogenic variants in high and moderate-risk genes, including *BRCA1* and *BRCA2*. Breast cancers is the most frequent cancer in *BRCA1/2* pathogenic variant carriers. Recently, studies showed *BRCA1* and *BRCA2* pathogenic variants carriers have a 72 and 69% cumulative risk up to age 80, respectively [4, 5]. Therefore, identifying more prognostic markers is required to screen high risk patients and it is significant for the development of effective therapeutic strategies.

Breast cancer susceptibility gene 1 (*BRCA1*) is a large gene with 23 exons located on chromosome 17q12.21. It plays as a tumor suppressor that is essential for maintenance of genome stability and DNA repair [6]. The most common mutation types of *BRCA1* are small insertion/deletion frameshift, nonsynonymous truncation, and splice-site alterations [7, 8]. Breast cancer risk based on *BRCA1* mutation carrier status will be greatly increased. Understanding the mutational spectra of *BRCA1* gene will help carriers to personalize the prevention strategies, including prophylactic mastectomy and salpingo-oopherectomy [9, 10]. The genetic testing for these mutations would directly affect the decisions of carriers and their family members.

In sporadic breast cancer, the frequency and relevance of *BRCA1*: p.Ile1845fs variant has not been elucidated completely. Therefore, we decided that a comprehensive investigation would be useful to clarify this mutation profiling and prognostic significance in sporadic breast cancer in Han Chinese. In the present study, the frequency of *BRCA1*: p.Ile1845fs mutation and the relationship between the mutation, clinicopathological parameters and clinical outcomes were evaluated.

## Material and methods

A total of 23,481 clinically high-risk breast cancer patients and 6489 healthy controls were recruited at 19 clinical centers in 11 Chinese provinces between 2012 to 2018. Clinicopathological features of the patients, including age, ethnicity, menopausal status, type of tumor, disease stage, lymph nodes and tumor size, were collected. Family history is defined that the breast cancer patients had one or more cancer patients (any kind of cancer) in the first-, second-, or third-degree relatives. The control subjects were hospital-based unrelated healthy individuals with no breast cancer or any other cancers. The written informed consents were signed by all participants. The study protocol was approved by the Ethics Committee of all the hospitals involved.

Genomic DNA was extracted from blood specimens using the QIAamp DNA kit (Qiagen). DNA were amplified by multiplex-amplicon PCR and libraries were then prepared using protocols recommended by Illumina. The validated DNA libraries were sequenced on an Illumina sequencing system (Illumina HiSeq X10). Read pairs (fastq data) generated from the sequencing system were aligned with reference sequences (*BRCA1*: NM_007300.3) and processed for variant calling. The pathogenic variant p.Ile1845fs was validated by sanger sequencing, and we successfully validated the mutation results with 100% concordance.

The statistical analysis were performed using the R program (http://www.r-project.org/). Chi Square test or the Fisher exact test were used to analyze the two-group comparisons and the OR and the corresponding 95% CI were estimated. All data were presented as the mean ± standard deviation (SD). *P*-values < 0.05 were considered statistically significant.

## Results

We analyzed the BRCA1 pathogenic variant, p.Ile1845fs, with breast cancer risk in 23,481 invasive breast cancer cases (46.24 ± 20.11 years) and age-matched 6489 controls (47.33 ± 13.46 years). A total of 94 p.Ile1845fs mutations were identified in 23,481 (0.40%) unselected breast cancer patients and 11 unaffected controls carried p.Ile1845fs mutation (0.17%, 11/6489). In the overall analysis, *BRCA1*: p.Ile1845fs variant showed a higher frequency in breast cancer cases (0.40%) than in controls (0.17%) with a greater than two-fold increased breast cancer risk (OR = 2.44, 95% CI = 1.12–5.34, *P* = 0.034, Table 1).

**Table 1:** BRCA1: p.Ile1845fs variant in unselected breast cancer cases and controls

Groups	Carriers	Non-carriers	Freq (%)	OR	95%CI	P
Controls	11	6489	0.17	2.36	1.26–4.89	**0.004**
Cases	94	23,481	0.40			

Bold: *P*<0.05

We summarized the clinicopathogical characteristics of the 94 patients with *BRCA1*: p.Ile1845fs variant and 23,387 non-carriers in Table 2. The mean age of these breast cancer patients was 46.16 years (sd = 9.80). The mean age of these non-carriers was 46.25 years (sd = 15.52). Among the 94 BRCA1: p.Ile1845fs variant carriers, 44 (46.81%) patients were diagnosed with estrogen receptor (ER) negative status. 46 (48.94%) patients were detected with progesterone receptor (PR) negative status. 35 (37.23%) patients presented with human epidermal growth factor receptor-2 (HER-2) negative status. 6 (6.38%) patients were classified with Luminal-A molecular typing. 26 (27.66%) patients were classified with Luminal-B molecular typing. 12 (12.77%) patients were classified with HER2 overexpression molecular typing. 19 (20.21%) patients were classified with TNBC (Triple-negative breast cancer) molecular typing. 35 (37.23%) patients had family history. TNBC molecular typing was more frequent in mutation carriers compared with non-carriers (*P* = 3.62E-6). *BRCA1*: p.Ile1845fs variant carriers were more likely to have family history of cancer (*P* = 0.034).

**Table 2:** Clinical characteristics of BRCA1: p.Ile1845fs variant carriers and non-carriers in this study

Variables	Carriers	Non-carriers	P
Age at diagnosis			0.19
≤ 50	52	6943	
> 50	22	4223	
na	20	12,221	
ER status			**2.21E-08**
Positive	24	11,849	
Negative	44	5622	
na	26	5916	
PR status			**1.29E-06**
Positive	21	10,630	
Negative	46	6794	
na	27	5963	
HER2 status			**0.011**
Positive	31	10,264	
Negative	35	6069	
na	28	7054	
Molecular typing			**3.62E-06**
Luminal-A	6	3726	
Luminal-B	26	8061	
HER2 overexpression	12	2805	
TNBC	19	1743	
na	31	7052	
Family history			
Yes	35	4184	**0.034**
No	41	8183	
na	18	11,020	
Total	94	23,387	23,481

Bold: *P*<0.05

## Discussion

In this study we investigated the profiling of the *BRCA1*: p.Ile1845fs variant in Han Chinese breast cancer. We conducted gene sequencing studies in 23,481 unselected breast cancer cases and 6489 controls and confirmed that *BRCA1*: p.Ile1845fs variant was associated with increased risk of breast cancer (OR = 2.36, 95%CI = 1.26–4.89, *P* = 0.004).

BRCA1 is a key factor in the DNA double-strand break repair of other genes that induce human cancers [11, 12]. It plays crucial roles in chromatin remodeling, cell-cycle regulation, and activating DNA repair in response to cellular stress [13, 14]. BRCA1 encodes a 1884-amino-acid-long nuclear protein (NP_009231.2) and is expressed in various tissues including breast tissues. There are more than 1600 known variants in *BRCA1* and its pathogenic variants increase the risks of breast cancer [15, 16]. Our genetic data suggested that *BRCA1*: p.Ile1845fs was a risk factor for breast cancer with statistically significant OR of 2.36.

Clinicopathological characteristics of *BRCA1*: p.Ile1845fs variant showed 44 (46.81%) patients were diagnosed with ER negative status, 46 (48.94%) with PR negative status and 35 (37.23%) with HER2 negative status. Based on estrogen receptor (ER), progesterone receptor (PR), and human epidermal growth factor receptor-2 (HER-2) status, we found 6 (6.38%) Luminal-A molecular typing patients and 26 (27.66%) Luminal-B molecular typing patients. Luminal A and Luminal B share similarities in prognosis, while Luminal B have lower expression of hormone receptors, higher expression of proliferation markers, and higher histologic grade than luminal A [17, 18]. Triple-negative breast cancer is defined by aggressive clinical behavior and occurs in 10–15% of sporadic breast cancers [19, 20]. There were 19 (20.21%) TNBC molecular typing patients carried p.Ile1845fs variant. Family history of breast or ovarian cancer is a high risk factor for breast cancer and genetic testing is recommend for these patients [21]. Among total 94 *BRCA1*: p.Ile1845fs variant carriers, 35 (37.23%) patients had family history.

Recently, more studies focus on effective detection of informative biomarkers for advanced development of early diagnosis and appropriate treatment in breast cancer. Arason A et al. showed the profiling of *BRCA1* c.4096 + 3A > G and found 8 heterozygous carriers (0.44%) in 1820 unselected breast cancer cases, and 3 (0.15%) in 1968 healthy controls [22]. *BRCA1*: p.Val1833Met variant was genotyped among 3531 breast cancer patients and 1558 healthy controls using sanger and next generation sequencing, with 27 (0.77%, 27/3531) carriers in cases while no carriers in controls [23]. Our study accord with a pathogenic *BRCA1* mutation: p.Ile1845fs and identified 94 carriers (0.40%) in 23,481 breast cancer patients, and 11 (0.17%, 11/6489) in controls. Our findings add to the current knowledge of *BRCA1*, which will be of use in clinical genetic counselling.

In summary, we described the frequency of *BRCA1*: p.Ile1845fs mutation and its clinical aspects in our cohort. We have found that *BRCA1*: p.Ile1845fs variant is associated with risk of breast cancer. Further genetic studies and meta-analyses are warranted to derive more precise risk estimates for *BRCA1*: p.Ile1845fs variant. And such carriers should be counselled accordingly, with clinical recommendations and personalized risk-reductionprimary and secondary cancer prevention strategies.

## Data Availability

The authors declare that the data supporting the findings of this study are available within the article.

## Abbreviations

BRCA1: Breast cancer susceptibility gene 1
ER: Estrogen receptor
HER-2: Human epidermal growth factor receptor-2
PR: Progesterone receptor
SD: Standard deviation
TNBC: Triple-negative breast cancer
